# Idiopathic calcinosis cutis universalis

**DOI:** 10.1186/1687-9856-2015-S1-P58

**Published:** 2015-04-28

**Authors:** Bina Akura, Lanny C Gultom

**Affiliations:** 1 Fatmawaty Hospital, Jakarta, Indonesia

**Keywords:** Calcinosis cutis, calsium phosphate, bisphosphonate, surgical excision

## Background

Calcinosis cutis is an uncommon disorder characterized deposition of crystals of calsium phosphate (hydroxyapatite) in the skin in various areas of the body. Medical and surgical treatments are options to cure calcinosis cutis. Because calcinosis cutis are not always well defined, a recurrence of the lesions may occur.

## Case

A 12-years old girl with complaints of multiple lumps on her body since 1 years prior to admission. Two years prior to admission patient complained of movement limitation due to pain when doing leg lifting, squatting and standing up. One years prior to admission patient got bilateral symmetrical lumps on hip, corn size and getting bigger. Two months prior to admission the lumps got ruptured and patient felt pain. From past history no calcium supplementation, no allergy.

In physical examination patient vital sign are within normal limits, moderate malnourished. From head and neck no enlargement of lymph nodes. Heart and lungs are normal. Abdomen is normal and no sign of edema on extremities. On the extremities multiple bilateral and symmetrical lumps, corn size and coin size. On the hip multiple bilateral and symmetrical lumps. The laboratory investigations revealed within normal limits. Biochemical examinations gave normal results for complete hemogram, erythrocyte sedimentation rate, blood sugar, uric acid, electrolyte, liver function and kidney function tests. Serum calcium 1.2 (normal 1.2 – 1.48 mg/dl), phosphorus 4.2 (normal 2.4-5.1 mg/dl), 25-hydroxyvitamin D 18.7(normal 17-54 ng/dl), parathormone (PTH) 29.46 (normal 15-65 pg/dl), and magnesium 2.0 mg/dl (normal 1.3-2.7 mg/dl).

On radiologic findings there are multiple calcification in soft tissue layer on humeral, antebrachial, femoral and crural bilateral. On tissue biopsy there are cystic space containing calcified material separated by fibrous tissue. Patient underwent treatment with bisphosphonate using zolendronate and surgical exicion

## Conclusions

Calcinosis cutis is an uncommon disorder which results in progressive deposition of insoluble calsium salts (crystals of calcium phosphate, hydroxyapatite) in the skin. Medical and surgical treatment are options to cure calcinosis cutis. Medical treatment using bisphosphonate. A better understanding of the process of calcinosis cutis will lead to therapies to improve patient morbidity.

Written informed consent was obtained from the patient for publication of this abstract and any accompanying images. A copy of the written consent is available for review by the Editor of this journal.

